# Blocking the Hepatic Branch of the Vagus Aggravates Hepatic Ischemia-Reperfusion Injury via Inhibiting the Expression of IL-22 in the Liver

**DOI:** 10.1155/2021/6666428

**Published:** 2021-05-08

**Authors:** Heng Zhou, Juling Xu, Sanxiong Huang, Ying He, Xiaowei He, Lu Guo, Shi Yin, Sheng Lu

**Affiliations:** ^1^Department of Pharmacy, The First People's Hospital of Huzhou, First Affiliated Hospital of Huzhou University, Huzhou 313000, China; ^2^Medical School of Huzhou University, Huzhou 313000, China; ^3^Department of Hepatobiliary Surgery, The First People's Hospital of Huzhou, Huzhou 313000, China; ^4^Zhejiang Provincial Key Laboratory of Media Biology and Pathogenic Control, Central Laboratory, First Affiliated Hospital of Huzhou University, Huzhou 313000, China; ^5^Department of Geriatrics, The First Affiliated Hospital of USTC, Division of Life Sciences and Medicine, University of Science and Technology of China, Hefei, Anhui 230001, China

## Abstract

Liver ischemia-reperfusion injury (IRI) is an inevitable process during liver transplantation, hemorrhagic shock, resection, and other liver surgeries. It is an important cause of postoperative liver dysfunction and increased medical costs. The protective effects of the vagus nerve on hepatic IRI have been reported, but the underlying mechanism has not been fully understood. We established a hepatic vagotomy (Hv) mouse model to study the effect of the vagus on liver IRI and to explore the underlying mechanism. Liver IRI was more serious in mice with Hv, which showed higher serum ALT and AST activities and histopathological changes. Further experiments confirmed that Hv significantly downregulated the expression of IL-22 protein and mRNA in the liver, blocking the activation of the STAT3 pathway. The STAT3 pathway in the livers of Hv mice was significantly activated, and liver injury was clearly alleviated after treatment with exogenous IL-22 recombinant protein. In conclusion, Hv can aggravate hepatic IRI, and its mechanism may be related to inhibition of IL-22 expression and downregulation of the STAT3 pathway in the liver.

## 1. Introduction

Hepatic ischemia-reperfusion injury (IRI) is an inevitable process during liver transplantation, hemorrhagic shock, resection, and other liver surgeries [1–4]. As a serious complication, ischemia-reperfusion-induced tissue injury accounts for about 10% of early graft failure after liver transplantation, and IRI is an important cause of postsurgery hepatic dysfunction [1, 3]. Unfortunately, current research on hepatic IRI is still insufficient, the mechanism has remained largely unclear [2, 3], and there is no recognized intervention for the treatment of hepatic IRI at present [3, 5, 6]. Thus, further study on the mechanism of hepatic IRI is necessary for the development of hepatic IRI therapy.

Interleukin-22 (IL-22), a cytokine discovered in 2000, plays a hepatoprotective role in different types of liver injury, including IRI [7–11]. While the role of cytokines and chemokines in hepatic IRI has been extensively investigated, some studies have also shown the role of the vagus nerve in liver IR injury [12, 13]. Zhang et al. [12] found that when the left cervical vagal trunk (the origin of the hepatic vagus nerve) was stimulated with a high-frequency electrode, the expression of nuclear factor erythroid 2-related factor 2 (Nrf2)/heme oxygenase-1 (HO-1) was markedly increased, which alleviated liver IRI by inhibiting oxidative stress, inflammation, and apoptosis in the liver. Another group established a hepatic vagotomy mouse model and showed that the vagus nerve could reduce IR-induced hepatocyte apoptosis by activating *α*7 nicotinic acetylcholine receptor (*α*7nAChR) on Kupffer cells (KCs) to prevent excessive reactive oxygen species production in KCs [13]. Vagus nerve signals activate macrophages and promote the release of IL-6, thus promoting liver regeneration after partial hepatectomy [14]. Therefore, the effect of the vagus on hepatocytes involves a variety of cytokines, chemokines, and signaling pathways, which we still know little about.

Herein, using a hepatic vagotomy plus IRI (Hv + IRI) mouse model, we investigated the role of the hepatic branch of the vagus in liver IRI and the potential mechanism of its effects. We found that liver injury was significantly aggravated after reperfusion in mice with vagus blockade. Further experiments showed that the expressions of IL-22 protein and IL-22 and IL-22 receptor-*α*1 (IL-22R*α*1) mRNA were significantly reduced, and the activation of the p-STAT3 signaling pathway was also inhibited in the livers of Hv mice after reperfusion. To determine the relationship between the decrease of IL-22 expression and the aggravation of hepatic IR injury after vagotomy, IRI-Hv mice were treated with exogenous IL-22 fusion protein. As expected, liver injury was significantly alleviated, and the p-STAT3 signaling pathway was clearly activated in the IL-22-treated mice, suggesting that the aggravation of liver injury induced by IR is related to the inhibition of IL-22 expression in the livers of mice that underwent hepatic vagotomy. These results provide a theoretical basis for using vagus nerve signaling as a new target for the treatment of liver diseases.

## 2. Materials and Methods

### 2.1. Materials

Recombinant mouse IL-22 was obtained from Novoprotein Scientific Inc. (Shanghai, China). Mouse anti-IL-22 antibody was purchased from R&D Systems (USA). Anti-STAT3, anti-phospho-STAT3 (Tyr 705), and anti-cyclinD1 antibodies were purchased from Cell Signaling Technology (USA).

### 2.2. Animals and Treatment

**Figure 1:**
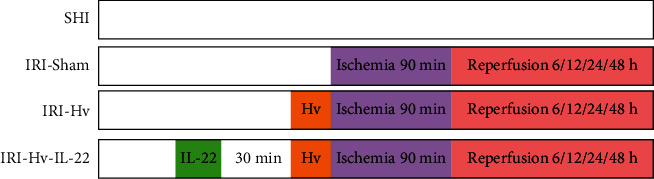
Schematic illustration of the experimental protocol. SHI: sham operation for hepatic ischemia-reperfusion injury; IRI-Sham: hepatic ischemia-reperfusion injury; IRI-Hv: hepatic ischemia-reperfusion injury + hepatic branch vagotomy; IRI-Hv-IL-22: hepatic ischemia-reperfusion injury + hepatic branch vagotomy + IL-22.

The male C57BL/6 mice (8–10 weeks old) used in this experiment were purchased from Beijing Vital River Laboratory Animal Technology Co. Ltd. (Beijing, China). The mice were forbidden to eat food but free to take water for 12 h before surgery. A midline laparotomy was performed in mice after anesthesia with pentobarbital sodium. The abdominal cavity was fully exposed and the vagus nerve of the hepatic branch was carefully dissociated and gently transected with fine forceps. Then, the bile duct, portal vein, and hepatic artery supplying the median and left liver lobes were clamped for 90 minutes. The mice were randomly divided into four groups: the SHI group, the IRI-Sham (hepatic ischemia-reperfusion injury) group, the IRI-Hv (hepatic ischemia-reperfusion injury + hepatic branch vagotomy) group, and the IRI-Hv-IL-22 (hepatic ischemia-reperfusion injury + hepatic branch vagotomy + IL-22) group. Mice in the IRI-Hv-IL-22 group were treated with IL-22 intravenously at 0.125 *μ*g/g body weight 30 min before the abdominal incision. Mice were euthanized at 6, 12, 24, and 48 h postreperfusion, and then serum and liver samples were collected for analysis (Figure 1).

### 2.3. Hepatic Branch Vagotomy

A midline abdominal incision was made to fully expose the abdominal cavity, pull down the esophagus and stomach, and pull up the right lobe and anterior lobe of the liver carefully to separate the hepatic branch of the vagus nerve from the ventral branch of the inferior vagus nerve a few millimeters above the cardia. For animals that underwent Hv, the vagus nerve of the hepatic branch was transected with fine forceps gently before the hepatic vessels were clamped.

### 2.4. Hepatic IRI Model

Partial hepatic ischemia-reperfusion (I/R) injury in mice was performed as previously described [15, 16]. In brief, C57BL/6 mice 8–10 weeks of age were anesthetized with pentobarbital sodium before a midline laparotomy was performed. Ligaments of the liver were dissected carefully, then the bile duct, portal vein, and hepatic artery supplying the median and left liver lobes were clamped with a vascular atraumatic clamp. After 90 min of ischemia, the clamp was removed, and the liver was reperfused. Mice were euthanized at 6, 12, 24, and 48 h postreperfusion, and then serum and liver samples were collected for analysis. Mice in the SHI group received identical treatment without vascular occlusion.

### 2.5. Analysis of Liver Injury

Serum and liver tissues were obtained after the mice were sacrificed. Serum alanine aminotransferase (ALT) and aspartate aminotransferase (AST) activity levels were measured by an automated chemical analyzer (Hitachi 3100, Japan) as important indexes to evaluate liver function. Liver tissue samples were fixed with formalin, embedded in paraffin, and cut into thin slices successively. Finally, the slices were stained with hematoxylin and eosin for pathological analysis.

### 2.6. Liver Weight/Body Weight Ratios (LW/BW)

Mice were weighed before euthanasia, then the whole liver was taken out and weighed. The ratio of liver weight to body weight (LW/BW) was counted as an indicator to reflect the degree of liver injury.

### 2.7. Measurement of IL-22 Serum Levels

Serum from mice was obtained at different time points postreperfusion as previously described. According to the manufacturer's instructions, serum IL-22 levels were quantified using commercial mouse ELISA kits (4A Biotech Co., Ltd., Beijing, China).

### 2.8. Quantitative RT-PCR Analysis

Total RNA from mouse livers was extracted using the TRIzol Reagent (Invitrogen; Thermo Fisher Scientific, Inc.), then reverse transcribed into cDNA using the PrimeScript Reverse Transcription Kit (Takara Biotechnology Co., Ltd.). SYBR Green PCR Master Mix (CoWin Biosciences) was used to perform quantitative PCR through a 7500 system (Applied Biosystems; Thermo Fisher Scientific, Inc.). 18sRNA was used as the housekeeping gene. The sequences of all the primers used in this study are listed in Table 1.

### 2.9. Immunohistochemistry

Methods of immunohistochemical staining of animal liver tissues followed standard protocols [3]. In brief, liver tissue sections were treated with 3% H_2_O_2_ and then blocked with 3% BSA after being deparaffinized with xylene and ethanol. Shaking off the blocking solution gently, the primary antibody for IL-22 (Abcam ab18498) was added dropwise on each slice and incubated overnight at 4°C. HRP-labeled secondary antibody was then added and incubated for 50 min at room temperature. After staining by 3,3′-diaminobenzidine, the number of positive cells was counted under a microscope. The numbers of IL-22^+^ hepatocytes and total hepatocytes in six microscope fields (×200) were counted, and the ratio of IL-22^+^ hepatocytes/total hepatocytes was calculated to directly determine the expression of IL-22 in liver tissues.

### 2.10. Western Blots

Western blotting analysis was performed to measure the level of protein expression in the liver as previously described [16]. The dilution ratios of the primary antibodies against IL-22 (0.1 *μ*g/mL), p-STAT3 (1 : 2000), STAT3 (1 : 1000), and cyclinD1 (1 : 1000) were based according to the manufacturers' protocols. Protein bands were visualized with a Hypersensitive Chemiluminescence Kit (Beyotime Institute of Biotechnology) using ImageJ v1.6.0 software.

### 2.11. Statistical Analysis

All data presented in this paper are expressed as means ± SD. Student's *t*-test was used for comparisons between two groups, and one-way ANOVA followed by Tukey's post hoc test was used when there were three or more groups. SPSS software (v19.0) was used for statistical analysis. Differences were considered statistically significant when *p* was less than 0.05.

## 3. Results

### 3.1. Hepatic Vagotomy Aggravates Hepatic Ischemia-Reperfusion Injury

**Figure 2:**
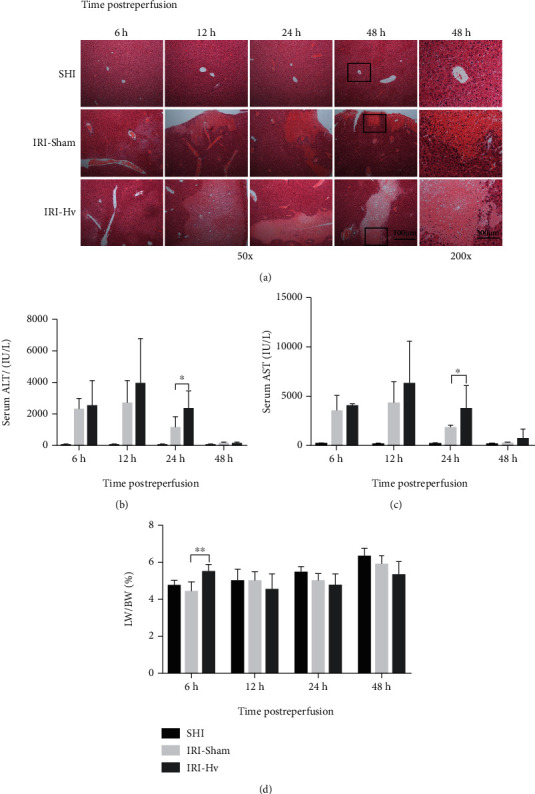
Hepatic vagotomy aggravates hepatic ischemia-reperfusion injury. (a) Representative H&E staining shows ischemic necrosis areas of liver tissues in the three groups at different time points (6 h, 12 h, 24 h, and 48 h) postreperfusion. Serum samples from different groups of mice were collected for ALT (b) and AST (c) activity detection. (d) LW/BW (liver weight/body weight) ratios of the three groups at different time points after reperfusion (*n* = 5‐7; ^∗^*p* < 0.05 and ^∗∗^*p* < 0.01).

Results of H&E staining showed that there were no obvious inflammatory areas or necrotic lesions in the livers of the SHI group, but instead, they had clear and orderly arranged tissue structures (Figure 2(a)). Necrotic foci with different shapes, sizes, and degrees were observed at different time points (6, 12, 24, and 48 h) after reperfusion in the IRI-Sham and IRI-Hv groups. Necrotic areas were mainly located at the edges of liver lobes, and inflammatory cell infiltration was observed at the edges of necrotic tissue. Compared with the IRI-Sham group, mice in the IRI-Hv group showed larger infarct sizes and more complete hepatocyte necrosis at different observation time points, indicating that the hepatic branch of the vagus has a protective effect on liver injury induced by ischemia-reperfusion.

Serum ALT and AST activities were significantly increased at 6–24 h postreperfusion, and reached peaks at 12 h in mice that suffered IRI. Compared with the IRI-Sham group, ALT activity of the IRI-Hv group at 6, 12, and 24 h and AST activity at 12, 24, and 48 h were higher, and this difference was statistically significant at 24 h (Figures 2(b) and 2(c)). Liver swelling was more obvious in the early stage postreperfusion (6 h) in mice that underwent Hv, and was characterized by increased LW/BW (Figure 2(d)). These data indicated that Hv aggravated the liver injury caused by ischemia-reperfusion.

### 3.2. Hepatic Vagotomy Reduces Hepatic IL-22 Production Induced by IRI

**Figure 3:**
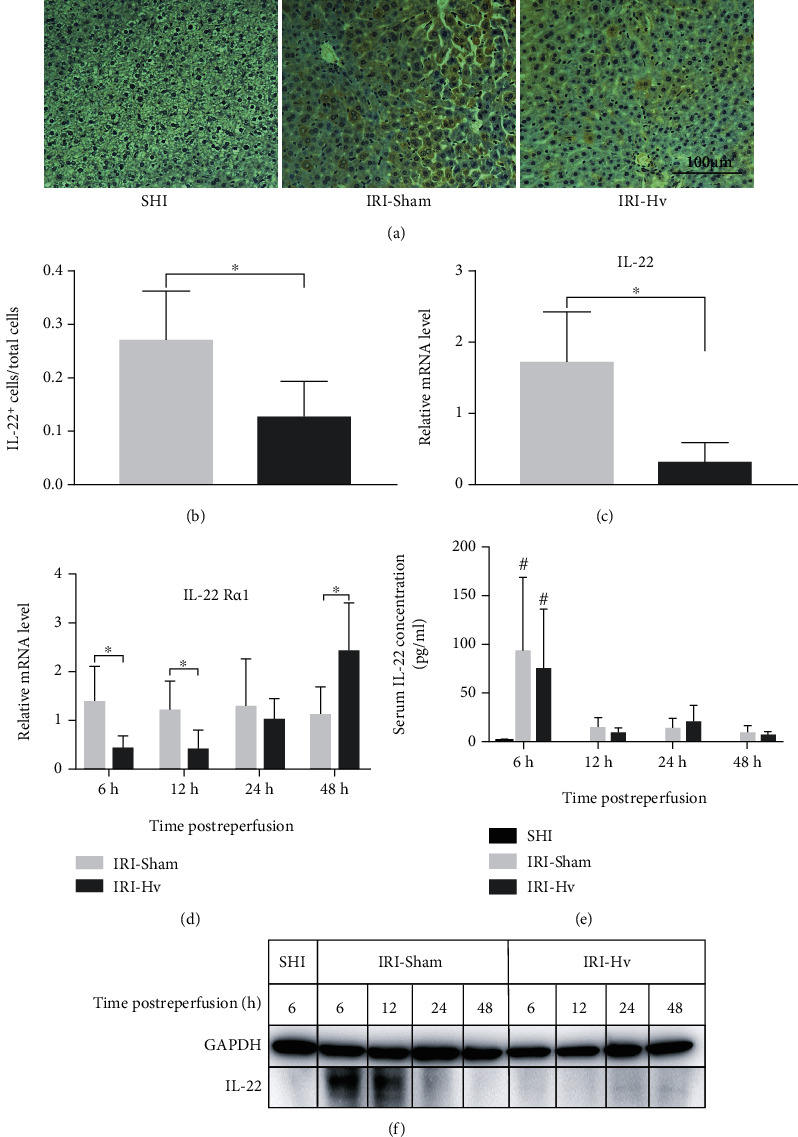
Hepatic vagotomy reduces hepatic IL-22 production induced by ischemia-reperfusion injury. (a) Typical immunohistochemical staining pictures of liver tissues in the three groups of mice at 12 h postreperfusion (×200 magnification). (b) The positive area of IL-22 staining is shown as brown, and the IL-22^+^ hepatocyte/total hepatocyte ratio is calculated. (c, d) Expressions of IL-22 (6 h) and IL-22R*α*1 mRNA in different liver tissues. (e, f) Serum IL-22 concentration and the expression of IL-22 protein in the livers of two groups at different time points after reperfusion (*n* = 4‐6; ^∗^*p* < 0.05. Compared with the SHI group: ^#^*p* < 0.05).

We observed that the serum IL-22 concentration of mice exposed to IRI increased significantly in the early stage (especially at 6 h) after reperfusion, which was significantly weakened thereafter (Figure 3(e)). This was also confirmed by western blotting for the IRI-Sham group (Figure 3(f)).

Immunohistochemistry, western blotting, and qPCR were used to determine the differences in IL-22 production between the IRI-Sham group and the IRI-Hv group. As expected, the IL-22^+^ hepatocyte/total hepatocyte ratio of the IRI-Sham group was significantly higher than that of the IRI-Hv group, which was confirmed by immunohistochemical staining (Figures 3(a) and 3(b)). Similarly, the expressions of IL-22 and IL-22R*α*1 mRNA in the IRI-Sham group were higher than those in the IRI-Hv group at different time points after reperfusion (Figures 3(c) and 3(d)). Compared with the SHI group, the expression of IL-22 protein in liver tissues of the IRI-Sham group increased significantly at 6 h, then began to decrease at 12 h, and could hardly be observed at 24 and 48 h. However, the expression of IL-22 protein was hardly seen at any time point in the IRI-Hv group (Figure 3(f)). These results suggest that Hv can inhibit the production of IL-22 induced by IRI. Although a huge difference in the expression of IL-22 was detected in liver tissues, there was no such difference in serum between the IRI-Sham group and the IRI-Hv group (Figure 3(e)).

### 3.3. Hepatic Vagotomy Reduces the Expression of P-STAT3 and CyclinD1 in the Liver

**Figure 4:**
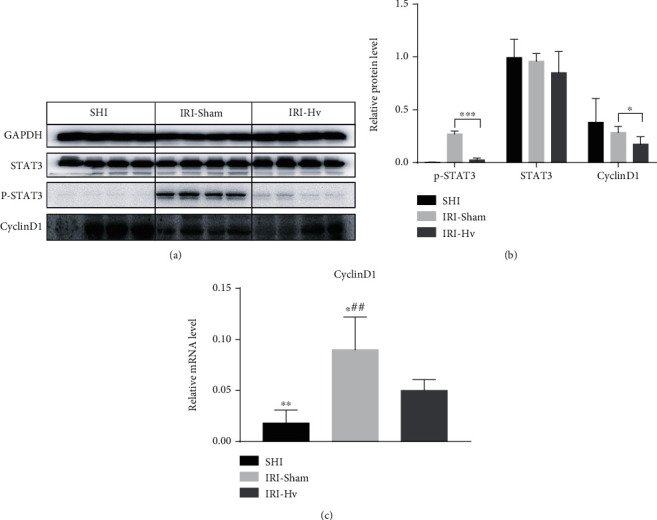
Effects of hepatic vagotomy on STAT3, p-STAT3, and cyclinD1 activation after reperfusion. (a, b) Western blotting was used to detect the expressions of STAT3, p-STAT3, and cyclinD1 proteins in livers of different groups (6 h). (c) Expression of cyclinD1 mRNA in different groups at 6 h after reperfusion (*n* = 4‐5; compared with the IRI-Hv group: ^∗^*p* < 0.05 and ^∗∗^*p* < 0.01; compared with the SHI group: ^##^*p* < 0.01).

The hepatoprotective function of IL-22 is achieved by activating the STAT3 pathway and inducing the expression of various promitogenic and antiapoptotic proteins. Previous studies have confirmed that IL-22 activates the STAT3 pathway and promotes the expression of cyclinD1 in livers damaged by different inducements, including IR [16]. Given that Hv reduced the expression of IL-22 in hepatocytes, we further tested the effect of this change on the activation of STAT3 and the expression of cell cycle proteins in hepatocytes. We performed western blotting and qPCR, and as expected, the expressions of p-STAT3 and cyclinD1 proteins and cyclinD1 mRNA were upregulated in the IRI-Sham group compared with the IRI-Hv group. Similarly, there were significant differences in p-STAT3 protein and cyclinD1 mRNA expressions between the IRI-Sham group and the SHI group (Figure 4).

### 3.4. Exogenous IL-22 Recombinant Protein Attenuates Liver Injury in IRI-Hv Mice

Further experiments were carried out to determine whether the aggravation of liver injury was related to the decrease of IL-22 expression induced by Hv in hepatocytes. Animals were randomly allocated into two groups: the IRI-Hv group and the IRI-Hv-IL-22 (hepatic ischemia-reperfusion injury plus hepatic branch vagotomy plus IL-22 treatment) group. Mice in the IRI-Hv-IL-22 group were treated with IL-22 intravenously at 0.125 *μ*g/g body weight 30 min before the abdominal incision, while the other group was given the same volume of PBS in the same way.

**Figure 5:**
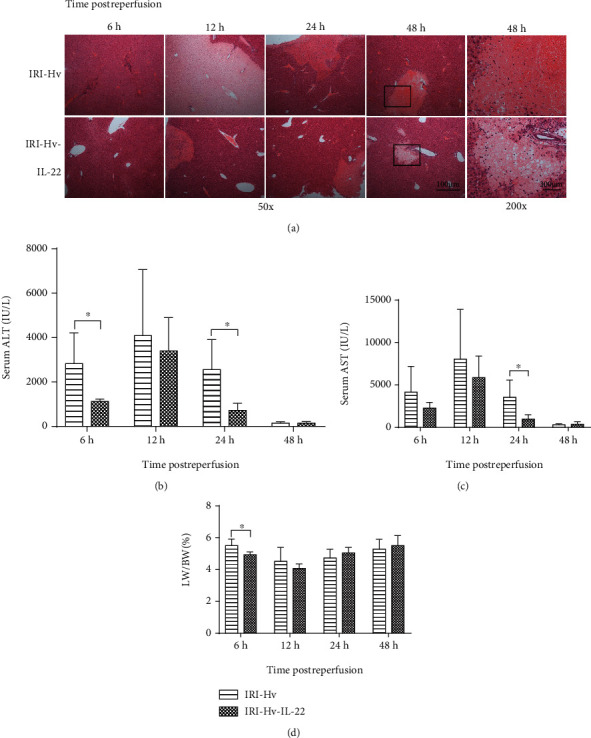
Administration of exogenous IL-22 recombinant protein reduces liver injury induced by IR. (a) H&E-stained liver sections show that the IRI-Hv group had larger infarct areas than the IRI-Hv-IL-22 group. (b, c) Serum ALT and AST concentrations in the IRI-Hv group were higher than those in the IRI-Hv-IL-22 group at several time points. (d) LW/BW ratios of the two groups at different time points after reperfusion (*n* = 4‐6, ^∗^*p* < 0.05).

Obvious necrotic foci and marginal infiltrating inflammatory cells were found in the livers of the IRI-Hv and the IRI-Hv-IL-22 groups, but the infarcted area of the former was larger (Figure 5(a)). We noticed that the serum ALT activity in the IRI-Hv group was higher than that in the IRI-Hv-IL-22 group at several time points, and it was statistically significant at 6 h and 24 h after reperfusion (Figure 5(b)). Similar to ALT, AST activity in the IRI-Hv-IL-22 group was lower than that in the IRI-Hv group postreperfusion, and the difference was significant at 24 h (Figure 5(c)). Compared with the IRI-Hv-IL-22 group, the LW/BW ratio in the IRI-Hv group was higher at 6 h, indicating that liver swelling in the IRI-Hv group was more serious (Figure 5(d)). In conclusion, these data suggest that IL-22 plays an important role in reducing liver injury in this model.

### 3.5. Exogenous IL-22 Recombinant Protein Promotes the Production of P-STAT3 and CyclinD1 in IRI-Hv Mice

**Figure 6:**
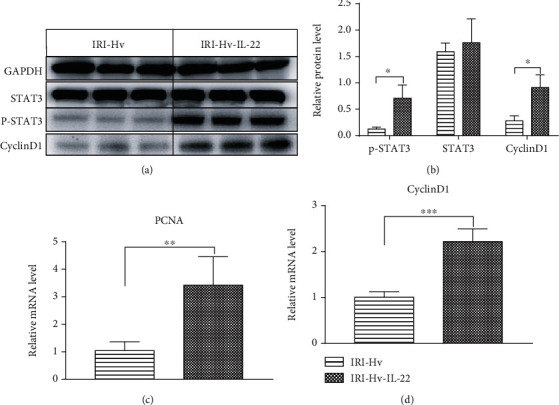
Exogenous IL-22 recombinant protein activates the hepatic STAT3 signaling pathway inhibited by Hv. (a, b) Western blotting results show that the expressions of p-STAT3 and cyclinD1 in the IRI-Hv-IL-22 group were significantly higher than those in the IRI-Hv group at 6 h. Similarly, we found a significant difference in the expression of cyclinD1 and PCNA mRNA between the two groups at 6 h by qPCR (c, d) (*n* = 4‐5, ^∗^*p* < 0.05).

The expressions of p-STAT3 and cyclinD1 protein and mRNA in livers were detected with western blotting and qPCR. Compared with the IRI-Hv group, the expression levels of p-STAT3 and cyclinD1 protein and proliferating cell nuclear antigen (PCNA) and cyclinD1 mRNA in the IRI-Hv-IL-22 group were higher, and the difference was statistically significant (Figure 6), indicating that treatment with exogenous IL-22 recombinant protein activated the STAT3 pathway inhibited by Hv in mice.

## 4. Discussion

In the current study, we showed that the liver injury induced by IR was significantly aggravated in mice undergoing Hv. Cutting off the vagus nerve increased the serum ALT and AST levels and clearly aggravated inflammatory cell infiltration and hepatocyte necrosis in livers after reperfusion. The mechanism may be related to the decrease of IL-22 expression and the inhibition of STAT3 signaling after the operation. This evidence suggests that the hepatic vagus nerve may play an important role in IR-induced liver injury through the IL-22-STAT3 axis.

Hepatic IRI involves a complex inflammatory pathway including Kupffer cell activation, leukocyte infiltration, and the release of proinflammatory cytokines, which eventually leads to hepatocyte damage and organ dysfunction [17–18]. Vagus nerve stimulation has been at the forefront of inflammatory disease research, and there have been many promising results [19–21]. Many studies have reported that the vagus nerve regulates the immune response effectively, a concept known as the cholinergic anti-inflammatory pathway [22–25]. The protective effect of vagus nerve signaling on IR injury in different organs of animals, including the myocardium and brain, has been reported [12, 26–27]. It has also been reported that the use of a high-frequency electrode to stimulate the vagus nerve during the whole course of IR can significantly reduce liver injury after reperfusion [12]. Lee et al. [22] found that treatment with nicotine (an acetylcholine receptor agonist) before ischemia had similar hepatoprotective effects in mice. To observe the effect of the vagus nerve on the liver more directly, we established a hepatic vagotomy mouse model. As expected, increased serum ALT and AST activities were attenuated by Hv in the IRI-Hv group. Histological H&E staining showed more inflammatory cell infiltration and hemorrhagic necrosis in the IRI-Hv group. These results are similar to those of previous reports showing protective effects of vagus nerve signaling on IR-induced liver injury [12, 22].

It has been reported that the expression of IL-22 is increased after liver injury [28–31]. The levels of IL-22 in the serum and liver mildly increase, while that of IL-22R*α* significantly increases after 70% hepatectomy in mice [28]. The expressions of IL-22 mRNA and protein are significantly increased in T cell-mediated hepatitis (induced by concanavalin A), but are lower in CCl4-induced liver injury in mice [29]. Serum levels of IL-22 in patients with compensated or stable decompensated liver cirrhosis are similar to those in healthy controls, but increase significantly in patients with acute decompensation of liver cirrhosis and acute-on-chronic liver failure (ACLF) [30]. The serum and intrahepatic levels of IL-22 are significantly increased in patients with drug-induced liver injury [31]. Therefore, the type (acute/chronic), stage, and severity of liver injury are important factors affecting the expression of IL-22 after injury. In this study, western blot analysis showed that the expression of IL-22 in the liver was increased in the early stage (6–12 h) of IR injury and was most significant at 6 h, then hardly observed after 24 h.

Vagus nerve stimulation (VNS) can control the release of T lymphocytes and then change the release of acetylcholine (Ach), which binds to *α*7 nicotinic acetylcholine receptors (*α*7nAChR) on macrophages and affects the release of many cytokines such as IL-1*β*, IL-6, and IL-18 [13, 19, 32–33]. Bernik et al. [34] confirmed that VNS inhibits the production of TNF then attenuates IR-induced shock. Another group has reported that vagus signaling upregulates the production of IL-6 by acting on hepatic macrophages [14]. Unfortunately, the effect of vagus nerve signaling on the expression of IL-22 in the liver is not fully understood. In our analysis, we found that, compared with the IRI-Hv group, the expressions of IL-22 protein and mRNA and IL-22R*α* mRNA in the IRI-Sham group were significantly increased, indicating that vagus nerve signaling plays an important role in the expression of IL-22 after liver injury.

At present, the potential mechanism of vagus nerve signaling in liver IRI is not fully understood. It has been reported that VNS significantly increases the protein level of Nrf2/HO-1 in the liver and reduces liver IRI by inhibiting inflammation, oxidative stress, and apoptosis [12, 22]. Another study found that VNS attenuates liver IRI by inhibiting TNF production [34]. One recent study reported the important role of the glutathione synthetase/glutathione/glutathione S-transferase signaling pathway in VNS-mediated liver protection against IRI [35]. We found that by inhibiting the expression of IL-22 in the liver, Hv reduced the activation of the STAT3 pathway, decreased the expression of antiapoptotic and mitogenic proteins, and aggravated the liver injury induced by IR. Treatment with exogenous IL-22 fusion protein reversed this injury by activating the STAT3 pathway. In conclusion, we confirmed that the hepatoprotective effect of the vagus nerve against IRI is closely related to the IL-22-STAT3 axis.

There are some limitations to this work. Although we observed the effect of Hv on IL-22 expression induced by IRI, the underlying mechanism remains unclear. There are many regulators that control the production of IL-22 *in vivo*, both positive (e.g., IL-23, IL-1*β*, and IL-7) and negative (e.g., IL-22BP, TGF-*β*, and IL-27) [36–38]. In addition, a wide variety of lymphoid cells can produce IL-22, covering both innate and adaptive immune system cells, including NKT cells, *αβ* T cells, innate lymphoid cells (ILCs), and *γδ* T cells [39–42]. At present, we do not know which targets the vagus signal acts on and how to regulate these targets to stimulate IL-22 production. The effect of IL-22 on liver regeneration has become a research focus in recent years, and whether the control of IL-22 production by vagus nerve signaling plays a key role in liver regeneration is also of great interest, so we will investigate these questions in the near future.

## 5. Conclusion

**Figure 7:**
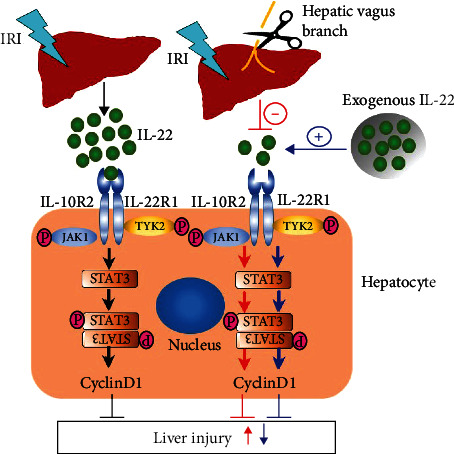
Schematic representation depicting the potential mechanisms of Hv (hepatic vagotomy) aggravating hepatic ischemia-reperfusion injury. Expression of IL-22 induced by IR injury in the liver was significantly downregulated when the hepatic vagus branch was cut off. Following this, the activation of STAT3 signaling pathway is downregulated, and then the expression of cyclin D1 is downregulated, which eventually aggravates liver injury. In mice treated with exogenous IL-22, the aggravating effect of Hv on liver injury was reversed due to the IL-22 supplementation.

We found that hepatic vagotomy can aggravate liver IRI, and the mechanism may be related to the downregulation of hepatic IL-22 expression, blocking the activation of the STAT3 pathway in the liver (Figure 7).

## Figures and Tables

**Table 1:** List of qPCR primer sequences.

Genes	Forward	Reverse
18S	5′-GTAACCCGTTGAACCCCATT-3′	5′-CCATCCAATCGGTAGTAGCG-3′
IL-22	5′-GCTCAGCTCCTGTCACATCA-3′	5′-CAGTTCCCCAATCGCCTTGA-3′
IL-22R*α*1	5′-TTACTACGCCAAGGTCACGG-3′	5′-GGCGGTTTGATGGTAGTGTG-3′
CyclinD1	5′-TCAAGTGTGACCCGGACTGC-3′	5′-CCTTGGGGTCGACGTTCTG-3′
PCNA	5′-CCTGTGCAAAGAATGGGGTG-3′	5′-TCTCTATGGTTACCGCCTCC-3′

## Data Availability

The data used to support the findings of this study are available from the corresponding authors upon request.
